# A genome-wide analysis of copy number variation in Murciano-Granadina goats

**DOI:** 10.1186/s12711-020-00564-4

**Published:** 2020-08-08

**Authors:** Dailu Guan, Amparo Martínez, Anna Castelló, Vincenzo Landi, María Gracia Luigi-Sierra, Javier Fernández-Álvarez, Betlem Cabrera, Juan Vicente Delgado, Xavier Such, Jordi Jordana, Marcel Amills

**Affiliations:** 1 Centre for Research in Agricultural Genomics (CRAG), CSIC-IRTA-UAB-UB, Universitat Autònoma de Barcelona, 08193 Bellaterra, Spain; 2 Departamento de Genética, Universidad de Córdoba, 14071 Córdoba, Spain; 3 Departament de Ciència Animal i dels Aliments, Facultat de Veterinària, Universitat Autònoma de Barcelona, 08193 Bellaterra, Spain; 4 Department of Veterinary Medicine, University of Bari “Aldo Moro”, SP. 62 per Casamassima km. 3, 70010 Valenzano, BA Italy; 5 Asociación Nacional de Criadores de Caprino de Raza Murciano-Granadina (CAPRIGRAN), 18340 Granada, Spain; 6 Group of Research in Ruminants (G2R), Department of Animal and Food Science, Universitat Autònoma de Barcelona (UAB), Bellaterra, Barcelona, Spain

## Abstract

**Background:**

In this work, our aim was to generate a map of the copy number variations (CNV) segregating in a population of Murciano-Granadina goats, the most important dairy breed in Spain, and to ascertain the main biological functions of the genes that map to copy number variable regions.

**Results:**

Using a dataset that comprised 1036 Murciano-Granadina goats genotyped with the Goat SNP50 BeadChip, we were able to detect 4617 and 7750 autosomal CNV with the PennCNV and QuantiSNP software, respectively. By applying the EnsembleCNV algorithm, these CNV were assembled into 1461 CNV regions (CNVR), of which 486 (33.3% of the total CNVR count) were consistently called by PennCNV and QuantiSNP and used in subsequent analyses. In this set of 486 CNVR, we identified 78 gain, 353 loss and 55 gain/loss events. The total length of all the CNVR (95.69 Mb) represented 3.9% of the goat autosomal genome (2466.19 Mb), whereas their size ranged from 2.0 kb to 11.1 Mb, with an average size of 196.89 kb. Functional annotation of the genes that overlapped with the CNVR revealed an enrichment of pathways related with olfactory transduction (fold-enrichment = 2.33, *q*-value = 1.61 × 10^−10^), ABC transporters (fold-enrichment = 5.27, *q*-value = 4.27 × 10^−04^) and bile secretion (fold-enrichment = 3.90, *q*-value = 5.70 × 10^−03^).

**Conclusions:**

A previous study reported that the average number of CNVR per goat breed was ~ 20 (978 CNVR/50 breeds), which is much smaller than the number we found here (486 CNVR). We attribute this difference to the fact that the previous study included multiple caprine breeds that were represented by small to moderate numbers of individuals. Given the low frequencies of CNV (in our study, the average frequency of CNV is 1.44%), such a design would probably underestimate the levels of the diversity of CNV at the within-breed level. We also observed that functions related with sensory perception, metabolism and embryo development are overrepresented in the set of genes that overlapped with CNV, and that these loci often belong to large multigene families with tens, hundreds or thousands of paralogous members, a feature that could favor the occurrence of duplications or deletions by non-allelic homologous recombination.

## Background

Copy number variations (CNV) encompass genomic deletions or duplications, with sizes ranging from 50 base pairs (bp) to several megabases (Mb), and which display polymorphisms (in terms of copy number) among individuals of a particular species [1–3]. In livestock, a broad array of phenotypes related with, among others, morphology [4, 5], pigmentation [6–9], sexual development [10] and susceptibility to disease [11] is caused by the segregation of CNV. Genome scans to detect structural variations in cattle have revealed that CNV regions (CNVR) are often enriched in genes that are involved in immunity [12–15], metabolism [12, 13], embryo development [12, 15] and sensory perception [13, 14]. There is evidence that the d_N_/d_S_ ratios of genes that map to taurine CNV are generally higher than those of genes that do not overlap with CNV, which indicates that CNV genes probably evolve under reduced selective constraint [13]. The analysis of gene networks has also shown that genes that co-localize with duplications tend to have fewer interactions with other genes than loci that do not overlap with CNV, reinforcing the idea that genes mapping to duplicated regions have fewer essential housekeeping functions than non-CNV genes, and also have reduced pleiotropy [13].

Although structural chromosomal variations can have strong effects on gene expression and phenotypic variability, technical limitations and the moderate quality of genome assemblies have hampered CNV mapping in livestock [1]. Until recently, this has been particularly true for goats. In 2010, Fontanesi et al. [16] published the first caprine CNV map by identifying, with the Bovine 385 k aCGH array, 127 CNVR including 86 and 41 copy loss and gain variants, respectively. Later on, resequencing the genome of individuals from several caprine breeds made it possible to identify CNV that overlap with 13 pigmentation genes and to detect an association between increased *ASIP* copy number and light pigmentation [17]. The first worldwide survey of copy number variation in goats was performed within the Goat ADAPTmap Project (http://www.goatadaptmap.org), and involved the genome-wide genotyping of 1023 goats from 50 breeds [18]. This study resulted in the identification of 978 CNVR among which several overlapped with genes that are functionally related with local adaptation such as coat color, muscle development, metabolic processes, and embryonic development [18]. Moreover, the patterns of the diversity of CNV differed according to geographic origin, which indicates that they have been influenced by population history [18]. In another study on 433 individuals from 13 East African goat breeds, Nandolo et al. [19] detected 325 CNVR. More recently, Henkel et al. [8] demonstrated the existence of complex patterns of structural variation in the regions containing the caprine *ASIP* and *KIT* genes, with potential causal effects on pigmentation. In spite of these efforts, the description of structural chromosomal variation in goats is still lagging behind that of other domestic species. Most of the CNV surveys in goats have analyzed large populations that represent a mixture of different breeds each with a limited number of individuals [18, 19], thus making it difficult to assess the magnitude of the CNV diversity at the within-breed level. Our goal was to fill this gap by analyzing a population of 1036 individuals from a single Spanish breed (Murciano-Granadina), and to investigate the functional roles of genes that map to CNVR and compare these results with data obtained in composite goat populations.

## Methods

### Genomic DNA extraction and high-throughput genotyping

Blood samples from 1036 Murciano-Granadina female goats from 15 farms that are connected through the use of artificial insemination were collected in EDTA K3 coated vacuum tubes and stored at − 20 °C before processing. Genomic DNA was isolated by a modified salting-out procedure [20]. Four volumes of red cell lysis solution (Tris–HCl 10 mmol/L, pH = 6.5; EDTA 2 mmol/L; Tween 20 1%) were added to 3 mL of whole blood, and this mixture was centrifuged at 2000×*g*. Pelleted cells were resuspended in 3 mL lysis buffer (Tris–HCl 200 mmol/L, pH = 8, EDTA 30 mmol/L, SDS 1%; NaCl 250 mmol/L) plus 100 µL proteinase K (20 mg/mL). The resulting mixture was incubated at 55 °C for 3 h followed by centrifugation at 2000×*g* in the presence of 1 mL of ammonium acetate (10 mol/L). The supernatant (~ 4 mL) was mixed with 3 mL of isopropanol 96%, which was subsequently centrifuged at 2000×*g* for 3 min. The supernatant was removed and the DNA pellet was washed with 3 mL of ethanol 70%. After centrifuging at 2000×*g* for 1 min, the DNA precipitate was dried at room temperature and resuspended in 1 mL of TE buffer (10 mmol/L Tris, pH = 8.0; 1 mmol/L EDTA, pH = 8).

High-throughput genotyping of the 1036 Murciano-Granadina DNA samples was carried out with the Goat SNP50 BeadChip [21] according to the manufacturer’s instructions (Illumina). Signal intensity ratios i.e. log R Ratio or LRR (the total probe intensity of a SNP referred to a canonical set of normal controls [22]), and B allele frequencies or BAF (relative quantity of one allele compared to the other one) [22], were exported for each single nucleotide polymorphism (SNP) with the GenomeStudio software 2.0.4 (Illumina, https://emea.illumina.com). Then, SNP coordinates were converted to the latest version of the goat reference genome (ARS1) [23]. After filtering out unmapped and non-autosomal SNPs and those with a call rate lower than 98%, a set of 50,551 SNPs remained for CNV mapping.

### Copy number variant calling with PennCNV and QuantiSNP

Based on their excellent performance in comparative studies, we selected two software packages, PennCNV v1.0.5 [24] and QuantiSNP v2 [25], to call CNV in the Murciano-Granadina population [26, 27]. The PennCNV software [24] detects CNV by applying the default parameters of the Hidden-Markov model. Population frequencies of B alleles were compiled based on the BAF of each SNP in the population. We used the “–gcmodelfile” option to adjust “genomic waves” [28]. The number of goat chromosomes was set with the “–lastchr 29” instruction. The QuantiSNP analysis [25] assumes an objective Bayes hidden-Markov model to improve the accuracy of segmental aneuploidy identification and mapping. This CNV calling software was run under default parameters by modifying the “–chr 1:29” option. The CNV that were supported by less than three SNPs were removed from the filtered set used here.

### Definition and functional annotation of copy number variant regions

We used the EnsembleCNV algorithm (beta version) [29] to assemble CNVR. All CNV called by PennCNV and/or QuantiSNP were combined to generate a set of initial CNVR by using the heuristic algorithm (threshold of minimum overlap = 30%) described in [29]. Subsequently, CNVR boundaries were refined by considering the local correlation structure of the LRR values of the SNPs mapping to CNVR [29]. Then, we reassigned the CNV calls that were initially obtained with PennCNV and QuantiSNP to each refined CNVR, so that the final set of CNVR comprised only those that were simultaneously detected by both callers. The resulting CNVR were matched to gene features that are annotated in the National Center for Biotechnology Information (NCBI, https://www.ncbi.nlm.nih.gov) by using BEDTools v2.25.0 [30]. In addition, we performed gene ontology (GO) enrichment and pathway analyses using the DAVID Bioinformatics Resources 6.8 [31, 32] based on human and goat background gene sets. The statistical significance was set to a *q*-value ≤ 0.05.

### Confirmation of copy number variant regions by quantitative real-time PCR

In order to evaluate the rate of false positives in our experiment, we conducted quantitative real-time PCR (qPCR) experiments to obtain an independent estimate of the copy number of four putative CNVR (CNVR_371_chr5, CNVR_506_chr6, CNVR_160_chr2 and CNVR_1229_chr21). Primers were designed with the Primer Express software (Applied Biosystems) to amplify specific regions of the *ADAMTS20*, *BST1*, *NCKAP5* and *TNFAIP2* genes (see Additional file 1: Table S1). As reference genes, we used the *melanocortin 1 receptor* (*MC1R*) and *glucagon* (*GCG*) genes (see Additional file 1: Table S1) loci [18, 33–35]. Quantitative PCR reactions contained 7.5 ng genomic DNA, 7.5 µL 2 × SybrSelect Master mix (Applied Biosystems), 4.5 pmol of each forward and reverse primer, and ultrapure water to a maximum final volume of 15 µL. Each sample was analyzed in triplicate in order to obtain averaged copy number estimates. Reactions were loaded onto 384-well plates and run in a QuantStudio 12 K Flex Real-Time PCR System instrument (Applied Biosystems). The specificity of the PCR reactions was evaluated with a melting curve analysis procedure, and the efficiency (96.2-105.4%) was assessed with standard curves. Thus, relative copy number was inferred with the qbase + software (Biogazelle, Ghent, Belgium) by using the 2^−ΔΔCt^ approach [36]. Copy number values were calibrated by taking as a reference, four samples which, according to Goat SNP50 BeadChip data, had two copies of the investigated genomic loci.

## Results

### Detection of copy number variation in Murciano-Granadina goats

**Fig. 1:**
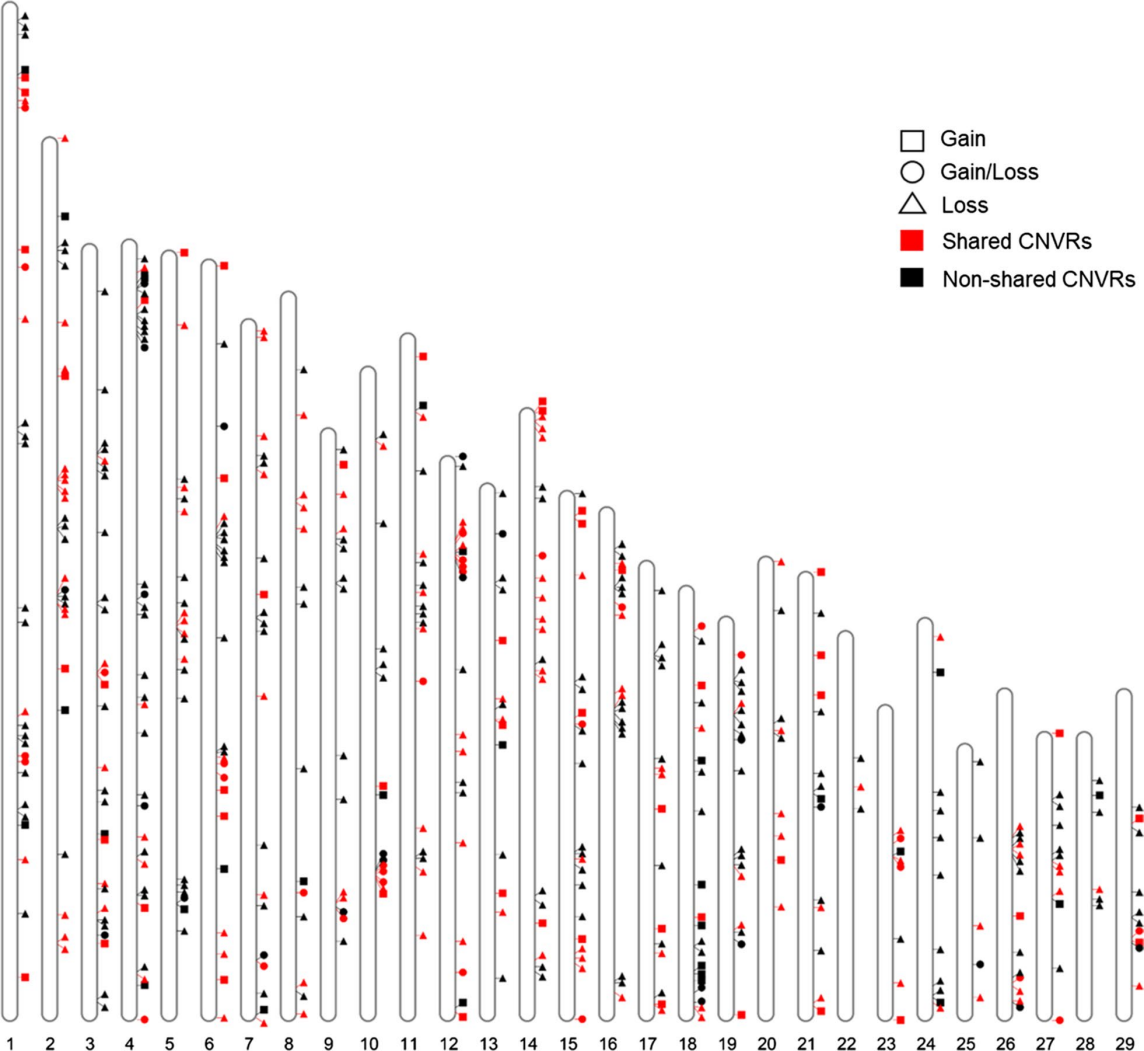
Genomic distribution of 486 CNVR detected with the PennCNV and QuantiSNP software on the 29 caprine autosomes. Squares, triangles and circles represent copy number gain, loss and gain/loss events, respectively. Red and black colors represent shared and non-shared CNVR, respectively. Shared CNVR are those detected both in our study and in Liu et al. [18], while non-shared CNVR are those identified only in our study

**Fig. 2:**
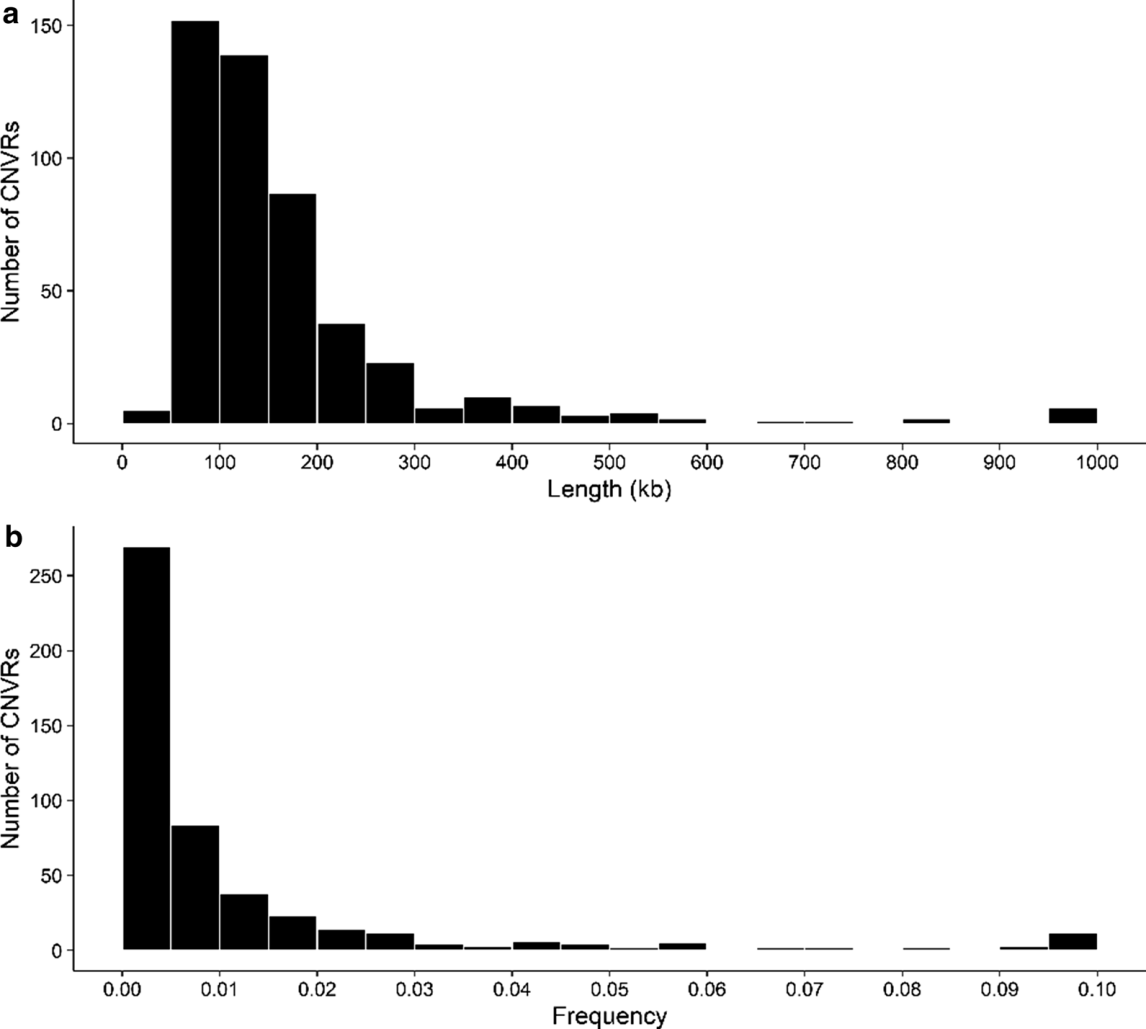
Histograms displaying the distribution of CNVR according to their size (**a**) and frequency (**b**). CNVR that were longer than 1000 kb were included in the 1000-kb bin, whereas those with frequencies above 0.1 were grouped in the 0.1 bin. The histograms were drawn by using the ggplot2 package (http://ggplot2.tidyverse.org/) implemented in R (https://www.r-project.org/)

The initial calling with PennCNV and QuantiSNP yielded 4617 and 7750 autosomal CNV, respectively. By using the EnsembleCNV tool [29], we assigned these CNV into 1461 CNVR with refined boundaries, of which 486 (33.3% of the total CNVR count) were detected simultaneously by PennCNV and QuantiSNP. The resulting CNVR included 78 copy gain, 353 copy loss and 55 copy gain/loss variants (Fig. 1, and Table 1) and (see Additional file 2: Table S2). The total length of the CNVR covered 95.69 Mb (3.9%) of the goat autosomal genome (2466.19 Mb), whereas their individual size ranged from 2.0 kb to 11.1 Mb, with an average of 196.9 kb (Fig. 2a and Table 1). Moreover, we found that 72.6% of the CNVR showed minimum allele frequencies lower than 0.01, with an average frequency of 1.44% (Fig. 2b). In addition, 10 CNVR with frequencies higher than 10% were distributed over seven caprine chromosomes. With a frequency of 41%, CNVR_1229_chr21 was the CNVR with the highest frequency in the whole dataset (see Additional file 2: Table S2). By using the BEDTools v2.25.0 program [30], 212 of the CNVR that we detected overlapped with 191 unique CNVR published by Liu et al. [18] (Fig. 1) and (see Additional file 2: Table S2). The CNVR that were detected in both studies are referred to as “shared CNVR”, whereas those that were identified in our study only are referred to as “non-shared CNVR” (Fig. 1). Six of the ten “shared CNVR” with frequencies higher than 0.1 show positional concordance with six CNVR detected by Liu et al. [18] (see Additional file 2: Table S2).

**Table 1:** Main features of copy number variation regions (CNVR) detected in 1036 Murciano-Granadina goats

Summary statistics	Total	Gain	Loss	Gain/loss
Total length (Mb)	95.69	26.52	61.17	8
Total number of CNVR	486	78	353	55
Number of CNVR (< 10 kb)	1	1	0	0
Number of CNVR (10–50 kb)	4	2	1	1
Number of CNVR (50–100 kb)	152	25	113	14
Number of CNVR (100–500 kb)	313	47	227	39
Number of CNVR (500 kb–1 Mb)	10	0	9	1
Number of CNVR (≥ 1 Mb)	6	3	3	0
Average number of SNPs per CNVR	5.59	9.01	5.03	4.35
Minimum size of CNVR (kb)	2.04	2.04	23.2	43.1
Maximum size of CNVR (kb)	11,124	11,124	1629.39	534.16
Average CNVR size (kb)	196.89	339.99	173.28	145.49
Standard deviation of CNVR size (kb)	539.35	1299.49	156.89	91.51

### Functional annotation of the genes that are located in copy number variable regions

Within the CNVR defined in our study, we detected 779 protein-coding genes according to the goat reference genome annotation (ARS1) [23] from the NCBI database (see Additional file 2: Table S2 and Additional file 3: Table S3). In a survey of the diversity of CNV in goats with a worldwide distribution, Liu et al. [18] detected 1437 copy number variable genes, of which 116 were also identified in our study and are referred to as “shared copy number variable genes” (see Additional file 3: Table S3). Among the “shared copy number variable genes”, the *ASIP* and *ADAMTS20* genes are particularly relevant: they are involved in pigmentation [6, 8, 17, 35, 37–39] and co-localize with selection signals detected in a worldwide sample of goats [40]. In addition, we found that about 11.4% (89) of the annotated genes that co-localize with CNVR are olfactory receptors or olfactory receptor-like genes (see Additional file 3: Table S3). Consistently, the most significantly enriched pathway was “Olfactory transduction” (*q*-value = 1.61 × 10^−10^, Table 2), followed by “ABC transporter” (*q*-value = 4.27 × 10^−4^, Table 2). A significant pathway related with immunity (i.e. Fc epsilon RI signaling, *q*-value = 0.02) was also identified based on a human background gene set (Table 2). Several overrepresented GO terms were related with embryonic skeletal system morphogenesis (*q*-value = 1.22 × 10^−3^) and G-protein coupled purinergic nucleotide receptor activity (*q*-value = 6.22 × 10^−3^, Table 2). Interestingly, the copy number variable genes were also enriched in pathways with metabolic significance, such as prolactin signaling and insulin signaling, as well as GO terms related with feeding behavior, but none of these pathways reached the significance threshold (*q*-value ≤ 0.05) after correction for multiple testing (see Additional file 4: Table S4). Several of the pathways outlined in Additional file 4: Table S4 play important roles in immunity (e.g. chemokine signaling, B cell receptor signaling and T cell receptor signaling), cancer (e.g. endometrial cancer, proteoglycans in cancer, thyroid cancer), as well as in oncogenic signaling (e.g. Ras and ErbB signaling) (see Additional file 4: Table S4), but most of them are not significant after correction for multiple testing.

**Table 2:** Functional enrichment of genes co-localizing with CNVR detected in 1036 Murciano-Granadina goats

Background gene set	Category	ID	Term	Number of genes	Fold enrichment	*P* value	*q*-value
Goat	KEGG	chx04740	Olfactory transduction	69	2.33	1.26E−11	1.61E−10
Goat	KEGG	chx02010	ABC transporters	11	5.27	3.33E−05	4.27E−04
Goat	KEGG	chx04976	Bile secretion	11	3.90	4.46E−04	5.70E−03
Human	KEGG	hsa04664	Fc epsilon RI signaling pathway	8	4.71	1.40E−03	1.76E−02
Human	GO/BP	GO:0009952	Anterior/posterior pattern specification	12	5.56	9.36E−06	1.61E−04
Human	GO/BP	GO:0048704	Embryonic skeletal system morphogenesis	8	7.60	7.13E−05	1.22E−03
Human	GO/BP	GO:0035589	G-protein coupled purinergic nucleotide receptor signaling pathway	5	13.24	4.18E−04	7.16E−03
Human	GO/CC	GO:0016020	Membrane	81	1.40	1.45E−03	1.98E−02
Human	GO/MF	GO:0003677	DNA binding	67	1.48	1.10E−03	1.60E−02
Human	GO/MF	GO:0045028	G-protein coupled purinergic nucleotide receptor activity	5	13.19	4.24E−04	6.22E−03

KEGG: Kyoto Encyclopedia of Genes and Genomes pathway; GO/MF: gene ontology (GO) term related with molecular function; GO/BP: GO term related with biological process; GO/CC: GO term related with cellular component

### Validation of four copy number variants by real-time quantitative polymerase chain reaction

**Fig. 3:**
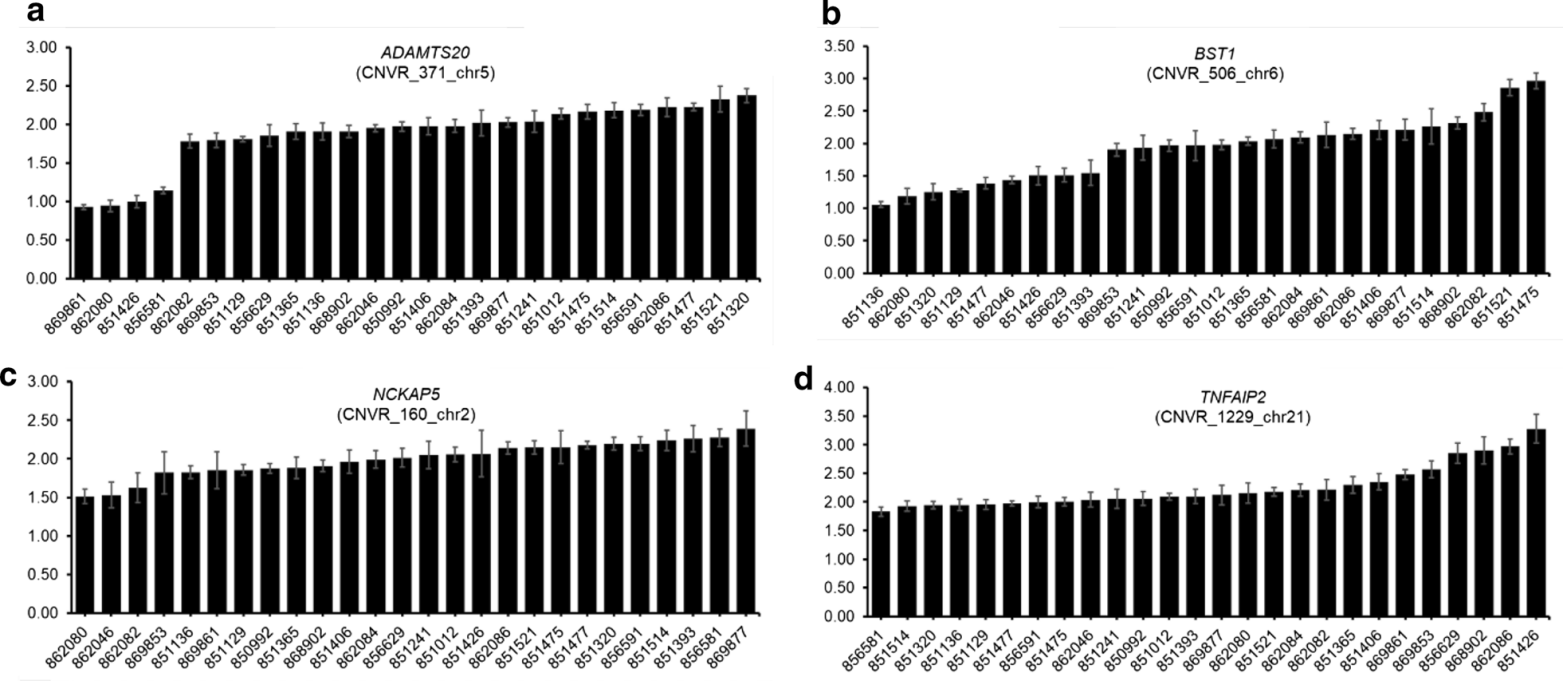
Relative quantification of four copy number variation regions by real-time quantitative polymerase chain reaction analysis: **a** CNVR_371_chr5 (*ADAMTS20*), **b** CNVR_506_chr6 (*BST1*), **c** CNVR_160_chr2 (*NCKAP5*), **d** CNVR_1229_chr21 (*TNFAIP2*). The *x* and *y* axes represent sample ID and relative quantification of CNVR (mean ± standard error, with each sample analyzed in triplicate), respectively. As calibrator, we used the average of four samples estimated to have two copies (diploid status) based on the Goat SNP50 BeadChip analysis

In order to confirm our results, we selected four CNVR (i.e. CNVR_371_chr5, CNVR_506_chr6, CNVR_160_chr2 and CNVR_1229_chr21) that co-localized with the *ADAMTS20*, *BST1*, *NCKAP5* and *TNFAIP2* genes, respectively (the primers used to amplify these CNVR are listed in Additional file 1: Table S1). As shown in Fig. 3, the estimated copy numbers obtained by qPCR analysis of Murciano-Granadina goat samples were: 0.93 to 2.38 copies relative to the calibrator (*ADAMTS20*), 1.06 to 2.96 copies (*BST1*), 1.51 to 2.39 copies (*NCKAP5*) and 1.83 to 3.28 copies (*TNFAIP2*). According to D’haene et al. [36], copy number estimates between 1.414 and 2.449 most likely correspond to a normal copy number of 2, whereas any number below or above these thresholds could represent a deletion or a duplication, respectively. Thus, based on these values, evidence of copy number variation was inferred for three of the four genes analyzed by qPCR.

## Discussion

In this work, our aim was to characterize copy number variation in Murciano-Granadina goats, a native Spanish breed used for milk production. By genotyping 1036 Murciano-Granadina goats with a SNP array, we were able to identify 486 CNVR covering 3.9% of the goat genome, whereas Liu et al. [18] identified CNVR that covered ~ 9% of the goat genome. The latter higher percentage reported by Liu et al. [18] can be explained by the fact that they analyzed 50 breeds with different geographical origins, i.e. a composite population that is probably much more diverse than that used in our work. Besides, the pipeline that we used to identify CNVR is more stringent than that employed by Liu et al. [18], removing CNVR that were not consistently detected by PennCNV and QuantiSNP. In the literature, estimates of 4.8 to 9.5% for CNVR coverage in the human genome are reported [2]. Our results and those obtained by Liu et al. [18] are consistent with these values.

Indeed, when Liu et al. [18] calculated the CNVR length for each breed normalized by the goat genome size, their results agreed well with our estimate of 3.9%. For instance, this parameter reached values of 3.94% in goats from Southeastern Africa and 3.13% in goats from Northwestern Africa and Eastern Mediterranean, whereas it was lowest (0.70%) for individuals from West Asia [18]. The number of CNV detected at the within-breed level by Liu et al. [18] was on average 126 CNV per breed and ranged from 6 to 714, whereas the average number of CNVR was only ~ 20 per breed [18]. Since the number of detected CNVR is proportional to population size, for most of the breeds investigated in [18], the level of within-breed CNV variation is probably underestimated. In summary, one important conclusion from our study is that the magnitude of CNV diversity at the within-breed level is likely to be much larger than that previously reported in studies that analyzed multiple populations, each represented by a small or moderate number of individuals.

Most of the CNVR that we report here ranged in size from 50 to 500 kb, with a mean size of 196.89 kb. Similarly, the average CNVR size reported by Liu et al. [18] was 268 kb. Both estimates are quite large and reflect that medium-density SNP arrays are not well suited to detect small CNVR in spite of their high abundance. In cattle, the average sizes of CNVR detected with the Illumina BovineHD Genotyping BeadChip (777 K SNPs) [14], Illumina whole-genome sequencing and PacBio sequencing [41] were 66.15, 10 and 0.81 kb, respectively. Another consistent feature of CNVR is that, in general, their frequencies are low or very low. In our study, approximately 73% of the CNVR had frequencies lower than 1% and the average frequency was 1.44%. Liu et al. [18] reported lower CNVR frequencies ranging from 0.34% (Alpine and Northern European goats) to 0.98% (Northwestern African goats). This decreased average CNVR frequency is not very significant and probably reflects differences in sampling size and the use of composite populations with multiple breeds, each one with its specific CNVR frequencies.

The CNVR detected in our study covered 779 protein-coding genes. Pathway analyses reflected a substantial enrichment of genes that are involved in olfactory perception, which is consistent with previous reports in cattle [13, 14]. In this regard, there is an important difference between our results and those by Liu et al. [18]. Whereas in the study of Liu et al. [18], the term “sensory perception” was underrepresented among the CNV genes (fold enrichment = 0.21), in our work the terms “olfactory transduction” (fold enrichment = 2.33) and “G-protein coupled purinergic nucleotide receptor activity” (fold enrichment = 13.19) were overrepresented, and many CNV genes were olfactory receptors. The two terms mentioned before are closely related because a broad array of purinergic receptors are differentially expressed in the olfactory receptor neurons that modulate odor responsiveness [42]. Moreover, purinergic nucleotides are important neuromodulators of peripheral auditory and visual sensory systems [42]. In cattle, Keel et al. [13] reported that “sensory perception of smell” and “G-protein coupled receptor signaling pathway” were significantly overrepresented in the protein-coding genes that overlapped with CNVR. Similarly, Upadhyay et al. [14] showed that “sensory perceptions of smell” and “chemical stimuli” are enriched in their set of CNV genes. A potential explanation for the underrepresentation of the “sensory perception” functional category among the genes overlapping CNV reported by Liu et al. [18] could be that in goats these genes are not well annotated yet, so the majority of them are identified with a LOC prefix and a number and, as a consequence of this, they are not correctly detected by PANTHER [43], thus biasing the results obtained in the gene ontology enrichment analysis.

Loci belonging to large multigene families might be more prone to co-localize with CNV because paralogous genes can act as templates in non-allelic homologous recombination events, which promote increases or reductions in copy number [44]. It should be noted that olfactory receptor genes constitute the largest gene superfamily, and in humans more than 900 genes and pseudogenes have been identified [45]. In cattle, 1071 olfactory receptor genes and pseudogenes are distributed in 49 clusters across 26 bovine chromosomes [46], and similar numbers have been reported for pigs [47]. Moreover, purifying selection against CNV is probably less intense in regions that contain olfactory-receptor genes than in genomic regions that contain genes with essential functions [48]. Interestingly, copy number changes in the olfactory receptor genes of wild and domestic mammals might have consequences on food foraging as well as on mate and predator recognition [49, 50].

In the set of genes that co-localize with CNVR, we also detected an enrichment of loci related with the multigene family of ATP binding cassette (ABC) transporters, a result that agrees well with previous findings in humans [51–54] and cattle [14, 56]. In mammals, ABC transporters fulfill the mission of carrying a broad array of endogenous substrates, such as amino acids, peptides, sugars, anions and hydrophobic compounds and metabolites across lipid membranes. At least 49 ABC genes that belong to eight subfamilies have been identified in the human genome [52]. Copy number variation in the human *ABCC4* and *ABCC6* genes is associated with susceptibility to esophageal squamous cell carcinoma [51] and to the rare autosomal recessive disease pseudoxanthoma elasticum [54], respectively. Moreover, large-scale deletions of the human *ABCA1* gene are a causative factor for hypoalphalipoproteinemia [53], a disease that is characterized by the complete absence of the apolipoprotein AI and extremely low levels of plasma high-density lipoprotein (HDL) cholesterol. We also found a highly significant enrichment of pathways related with embryo development (anterior/posterior pattern specification, embryonic skeletal system morphogenesis), as previously reported [18]. These pathways are featured by genes that belong to the Hox multigene family of transcription factors, possibly reflecting the genomic instability of certain homeobox gene clusters as evidenced by the existence of many synteny/paralogy breakpoints and assembly gaps as outlined in comparative studies [55].

Although not significant after correction for multiple testing, we detected an enrichment of pathways with metabolic significance, such as prolactin and insulin signaling, which could have an impact on milk production and growth [57–59]. Interestingly, the comparison of our work with that of Liu et al. [18] revealed 116 protein-coding genes that co-localize with the set of shared CNVR. One of the most relevant shared genes encodes ASIP, a protein that increases the ratio of pheomelanin to eumelanin by binding to the melanocortin 1 receptor and delivering an antagonist signal that blocks the downstream expression of eumelanogenic enzymes [60]. Mutations in the *ASIP* gene play critical roles in animal pigmentation [39]. For instance, the causal factor of the white color typical of many sheep breeds is the ubiquitous expression of a duplicated copy of the *ASIP* coding sequence, which is regulated by a duplicated promoter corresponding to the *itchy E3 ubiquitin protein ligase* gene [6, 39]. Although some studies proposed that the *ASIP* CNV might be associated with different pigmentation patterns in goats [8, 17, 37], no functional assay has verified an association of *ASIP* copy number with *ASIP* mRNA levels. Another interesting shared copy number variable gene is *ADAMTS20*, which was also identified in two previous CNV surveys [17, 18]. This gene encodes a metalloproteinase with an important role in melanoblast survival by mediating Kit signaling [38] and in palatogenesis [61]. Bertolini et al. [40] performed a selection scan in white vs. colored (black and red) goats and detected a selective sweep in the *ADAMTS20* gene. In the light of these results, the potential involvement of a structural variation in *ADAMTS20* in goat pigmentation should be explored further. Moreover, it is worthwhile to mention that several CNVR genes have functions related with production and reproduction traits. For instance, the *NCKAP5* gene, which co-localizes with CNVR_160_chr2 (frequency = 0.1), is associated with milk fat percentage in cattle [62]. Taking the above evidence into account, the implication of structural chromosomal variations in the genetic determinism of traits of economic interest with a complex inheritance deserves further exploration by designing tools that allow inferring CNVR genotypes with high confidence.

## Conclusions

With the PennCNV and QuantiSNP software, we detected 486 CNVR in the genome of the Murciano-Granadina breed. In a previous study [18] that used a less stringent pipeline (only PennCNV was used) and included multiple populations with small to moderate sample sizes, the average number of CNVR events per breed was ~ 20. One conclusion of our study is that CNV surveys, which are based on a broad array of breeds represented by only a few individuals, underestimate the true levels of the CNV diversity at the within-breed level. The main reason for this outcome is that since the majority of CNV have very low frequencies, they cannot be detected efficiently when sample size is small and, in consequence, much of the existing variation is missed. We have also found that genes that overlap with CNV are functionally related with olfactory transduction, embryo development, ABC transporters and G-protein coupled purinergic nucleotide receptor activity. Most of these genes belong to large multigene families encompassing tens, hundreds or thousands of paralogous genes that could act as substrates in non-allelic homologous recombination events, which is one of the main mechanisms generating duplications and deletions in humans and other species. Finally, we detected CNV that co-localize with the *ASIP* and *ADAMTS20* pigmentation genes, which according to previous studies have been subjected to positive selection for coat color in goats.

## Supplementary information

**Additional file 1: Table S1.** List of primers used in the real-time quantitative PCR experiment to validate four putative copy number variable genes.

**Additional file 2: Table S2.** List of copy number variation regions (CNVR) consistently detected with PennCNV and QuantiSNP in 1036 Murciano-Granadina goats.

**Additional file 3: Table S3.** List of copy number variable genes detected in the current work and their concordance with those reported by Liu et al. [18].

**Additional file 4: Table S4.** Functional enrichment of genes co-localizing with copy number variation regions detected in 1036 Murciano-Granadina goats.

## Data Availability

The dataset supporting the conclusions of this article is accessible at Figshare (10.6084/m9.figshare.12674357).
